# Prediction of early recurrence and response to adjuvant Sorafenib for hepatocellular carcinoma after resection

**DOI:** 10.7717/peerj.12554

**Published:** 2021-11-26

**Authors:** Liming Zheng, Xi Gu, Guojun Zheng, Xin Li, Meifang He, Longgen Liu, Xike Zhou

**Affiliations:** 1 Central Laboratory, The Third People’s Hospital of Changzhou, Changzhou, China; 2 Department of General Surgery, The Third People’s Hospital of Changzhou, Changzhou, China; 3 Clinical Lab, Wuxi No. 5 People’s Hospital, Wuxi, China

**Keywords:** Hepatocellular carcinoma, Early recurrence, Adjuvant sorafenib

## Abstract

**Background:**

Early recurrence of hepatocellular carcinoma (HCC) is a major obstacle to improving the prognosis, and no widely accepted adjuvant therapy guideline for patients post-liver resection is available. Currently, all available methods and biomarkers are insufficient to accurately predict post-operation HCC patients’ risk of early recurrence and their response to adjuvant therapy.

**Methods:**

In this study, we downloaded four gene expression datasets (GSE14520, GSE54236, GSE87630, and GSE109211) from the Gene Expression Omnibus database and identified 34 common differentially expressed genes associated with HCC dysregulation and response to adjuvant sorafenib. Then, we constructed a novel 11-messenger RNA predictive model by using ROC curves analysis, univariate Cox regression analysis, and LASSO Cox regression analysis. Furthermore, we validated the predictive values of the risk model in GSE14520 and TCGA-LIHC cohorts by using Kaplan–Meier survival analysis, multivariable Cox regression analysis, and decision curve analysis, respectively.

**Results:**

The risk score model could identify patients with a high risk of HCC recurrence at the early stage and could predict the response of patients to adjuvant sorafenib. Patients with a high risk score had a worse recurrence rate in training cohorts (2-year: *p* < 0.0001, hazard ratio (HR): 4.658, confidence interval 95% CI [2.895–7.495]; 5-year: *p* < 0.0001, HR: 3.251, 95% CI [2.155–4.904]) and external validation cohorts (2-year: *p* < 0.001, HR: 3.65, 95% CI [2.001–6.658]; 5-year: *p* < 0.001, HR: 3.156, 95% CI [1.78–5.596]). The AUC values of the risk score model for predicting tumor early recurrence were 0.746 and 0.618, and that of the risk score model for predicting the response to adjuvant sorafenib were 0.722 and 0.708 in the different cohort, respectively. Multivariable Cox regression analysis and decision curve analysis also showed that the risk score model was superior to and independent of other clinicopathologic characteristics. Moreover, the risk score model had excellent abilities to predict the overall survival and HCC recurrence of patients with the same tumor stage category.

**Conclusions:**

Our risk model is a reliable and superior predictive tool. With this model, we could optimize the risk stratification based on early tumor recurrence and could evaluate the response of patients to adjuvant sorafenib after liver resection.

## Introduction

Hepatocellular carcinoma (HCC), as one of the most common tumors in the world with an extremely poor prognosis, is the third leading cause of cancer-related death (Bray et al., 2018). Despite recent improvements in potential curative therapies, the prognosis of patients with HCC is still far from satisfactory due to tumor recurrence. Evidence suggests that the 3-year recurrence rate of HCC in patients treated with resection or radiofrequency ablation is about 50% and the 5-year recurrence rate is up to 70% (Llovet et al., 2016). This high risk of HCC recurrence severely compromised the overall survival (OS) of the patients (Lu, Cheng & Poon, 2014). Therefore, having effective prevention of HCC recurrence after curative treatments, including the clearance of tumor micro-metastasis and the adjuvant treatments for the *de novo* tumors, is a great unmet medical need. Meanwhile, figuring out reliable models and biomarkers for accurately predicting tumor recurrence is also urgently needed.

In the past decade, many adjuvant therapies have been proposed or applied to HCC patients, such as chemotherapy (Li et al., 2020b), chemoembolisation (Wang et al., 2018), or immune therapies (Lee et al., 2015). Unfortunately, most of them are not recommended in clinical practice due to having relatively low efficacies or getting inconclusive results (European Association for the Study of the Liver, 2018; Heimbach et al., 2018). As a Tyrosine Kinase Inhibitor (TKI) agent that suppresses tumor proliferation and angiogenesis, sorafenib has been approved by the US Food and Drug Administration (FDA) as the first-line treatment for advanced HCC (Wilhelm et al., 2006). One study demonstrated that sorafenib has clinical benefits in patients with hepatectomy (Li et al., 2020a), but another one showed that the benefits were negligible (Bruix et al., 2015). Therefore, it is necessary to identify the patients, who would benefit from the adjuvant sorafenib after liver resection. Sorafenib is an oral multiple tyrosine kinases inhibitor that blocks the proliferation and angiogenesis of tumor cell (Wilhelm et al., 2008). Despite multiple studies have been done in patients with HCC, no single biomarker that can clearly predict the response of patients to sorafenib has been identified yet. Therefore, a combination of multiple biomarkers is needed to predict the response of patients to sorafenib.

Generally, the risk of tumor recurrence within 2 years after resection, which is categorized as early recurrences and is accounting for more than 70% of tumor recurrence, is associated with the aggressiveness of the primary tumor (Chan et al., 2018); while late recurrence, which is defined as tumor recurrence 2 years after resection, are usually thought to be due to *de novo* carcinogenesis or underlying liver disease (Portolani et al., 2006). Therefore, being able to identify patients with a high risk of early recurrence after hepatic resection can provide a reference for clinicians to detect the recurrence of HCC as early as possible and to conduct adjuvant therapies. Although the association between individual molecular markers and early recurrence in HCC has been reported in several literatures, the clinical utility of these markers was limited because of the heterogeneity of HCC (Friemel et al., 2015). Traditional diagnosis methods (such as serological biomarkers, tumor staging) still have technological limitations for predicting accurately recurrence (Iizuka et al., 2003). Ogle reported that integrating multiple parameters into a single model would greatly improve the clinical utility compared with a single biomarker (Ogle et al., 2016). However, models that predicted early recurrence of HCC and response to adjuvant sorafenib were rarely found. Therefore, in the present study, based on the gene expression data of HCC, we attempted to construct and validate a multi-mRNA signature risk model that could effectively predict HCC early recurrence and could evaluate clinical benefits of adjuvant sorafenib in patients, who received resection, using an integrated bioinformatics method.

## Materials and Methods

### Datasets of HCC

We carefully screened four gene expression datasets (GSE14520, GSE87630, GSE54236, and GSE109211) with a total of 504 HCC patients from Gene Expression Omnibus (GEO, http://www.ncbi.ncbi,nlm.nih.gov/geo) and downloaded Liver Hepatocellular Carcinoma (LIHC) dataset containing 219 patients with relapse-free survival (RFS) status from The Cancer Genome Atlas (TCGA, https://portal.gdc.cancer.gov). In our research, the datasets from GEO were used as the discovery cohorts, while TCGA-LIHC was taken as the validation cohort. Among the discovery cohorts, three datasets (GSE14520, GSE87630, and GSE54236) were acquired on different sequencing platforms (Affymetrix, Illumina, and Agilent) and different sample backgrounds (America, Korea, and Italy). GSE109211 dataset included 140 patients with hepatic resection, of whom 67 patients received sorafenib as adjuvant therapy and 73 patients with Placebo. In addition, the complete clinical prognostic information of patients in GSE14520 and LIHC-TCGA were downloaded, respectively.

### Identification of differentially expressed genes

The online analytical tool of GEO2R (https://www.ncbi.nlm.nih.gov/geo/geo2r) was used to identify Differentially Expressed Genes (DEGs) between tumor and normal tissues, and the volcano plots of genes were visualized using the R package ‘ggplot2’. Genes with fold change (FC) > 1.5 and adjusted *p-*value < 0.05 were reserved as DEGs (Ritchie et al., 2015). Furthermore, Venn diagram analyses were performed with R package ‘VennDiagram’ to obtain the overlapping DEGs, and the overlapping DEGs within all series were retained as credible DEGs. Besides, Functions Enrichment Analyses (GO-biological processes) and the clustering analysis of the credible DEGs were respectively performed using the R package ‘clusterProfiler’ and ‘pheatmap’.

### Establishment and validation of LASSO Cox regression model

To identify DEGs related to early relapse (within 2 years after resection) in GSE14520, we used the Youden index method in receiver operating characteristic (ROC) analysis. According to the cutoff values, the expression level of each gene was converted into binary status (low expression status was equal to 0 and high expression status was equal to 1). Based on the binary status data of each gene, the univariate Cox regression analysis were conducted to further select the early relapse related DEGs. The predicted time was equal to 24 months, and the criteria were as follows: the area under the ROC curve (AUC) > 0.55 and *p*-value < 0.25, respectively. Finally, these selected DEGs with the binary status were further analyzed using the least absolute shrinkage and selection operator (LASSO) Cox regression which could be used to construct risk models by screening variables in high dimension data (Gui & Li, 2005; Shi et al., 2017). According to 10-fold likelihood cross-validation and ran up to 100 times, the most powerful prognostic genes were identified and the risk score model was constructed. The model = $\; \mathop \sum \nolimits_{i = 1}^n coeffcient*Expression\; status\; of\; DEGs\; \left( i \right)$. Based on this formula, patients were divided into high- or low-risk groups according to the optimal cutoff risk score value determined by ROC for predicting tumor early recurrence. Next, ROC curves were applied to evaluate the accuracy of the risk score model for predicting tumor early recurrence and response to sorafenib. Kaplan–Meier method and Log-Rank test were carried out to compare the recurrence and OS rate between two groups. Univariate and Multivariate Cox regression analyses (Zhang et al., 2018a) were performed for potential prognostic factors such as genders (female *vs*. male), metastasis (high *vs*. low), cirrhosis (yes *vs*. no), clinicopathological features (TNM, BCLC, CLIP stage) and risk score model. Furthermore, decision curve analysis (DCA), which was widely used to measure the clinical utility of specific models was performed (Vickers et al., 2008; Zhang et al., 2018b). The advantage of DCA is that it integrates the preferences of patients or policy-makers into analysis. If the net benefit of a model is greater than treating all and none patients, the model has clinical utility.

### Statistical analysis

Kaplan–Meier plots and log-rank tests were performed using GraphPad Prism (version 7.0). And ROC curves, Univariable or multivariable Cox regression analyses, LASSO Cox regression, DCA plots and Calibration curve analysis were performed using R (version 3.6.1) packages ‘pROC’, ‘survival’, ‘glmnet’, ‘devtools’, ‘DecisionCurve’, ‘rmda’, respectively. The values of *p* < 0.05 in all tests were considered statistically significant.

## Results

### Identification and analysis of differentially expressed genes in HCC

**Figure 1:**
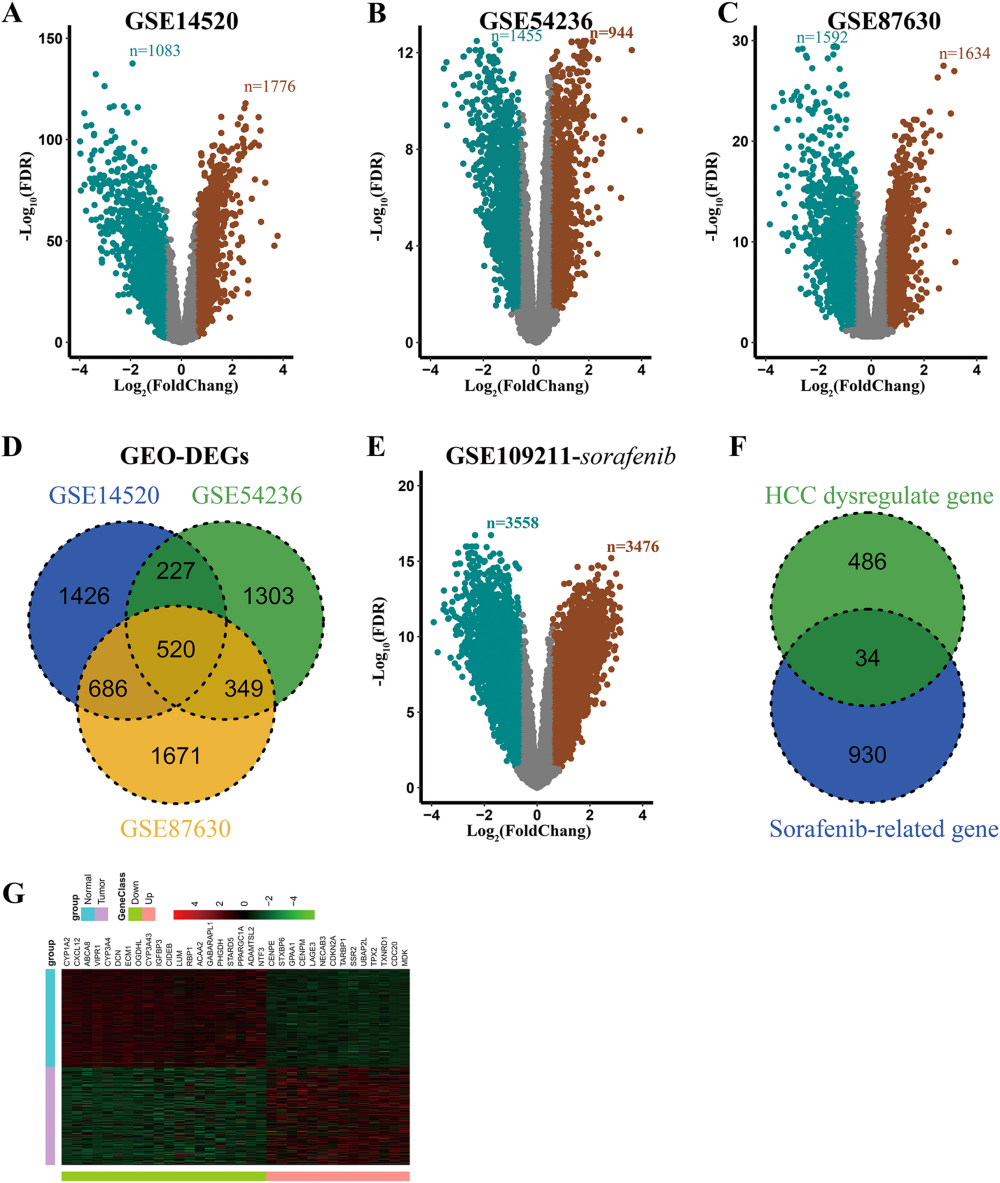
Identification of DEGs associated with response to adjuvant sorafenib. (A–C) Volcano plots of genes in selected datasets (GSE14520, GSE54236, and GSE87630). SaddleBrown: up-regulated DEGs; Teal: down-regulated DEGs. (D) Overlaping Analysis of DEGs in three datasets. (E) Volcano plots of genes related to sorafenib in GSE109211 dataset. (F) Overlaping Analysis of dyregulated and sorafenib related genes in HCC. (G) Heat map of 34 overlapping DEGs in GSE14520 dataset.

Detailed information of all eligible datasets as described in Table 1. After analyzing these datasets using GEO2R, 2859, 2399, and 3226 DEGs were obtained and were displayed by volcano plots (Figs. 1A–1C). Then the overlapping analysis of DEGs in three eligible datasets (GSE14520, GSE87630, and GSE54236) was conducted, and a commonly dysregulated gene set containing 520 genes in HCC was obtained (Fig. 1D). Similarly, in the GSE109211 dataset, 7034 and 6874 DEGs were obtained from the adjuvant treatment group with sorafenib and the placebo group, respectively (Figs. 1E and S1A). Meanwhile, the setdiff function analysis of DEGs in GSE109211 showed that 964 genes were associated with sorafenib adjuvant treatment but not with placebo (Fig. S1B). Finally, 34 overlapping DEGs between HCC-related dysregulate genes and sorafenib-related genes were identified, which were considered to be commonly dysregulated in HCC and were related to the response to adjuvant sorafenib in patients, who received liver resection (Fig. 1F). Clustering analysis of these genes was conducted by a heat map, including 14 up-regulated and 20 down-regulated genes (Fig. 1G).

**Table 1:** GEO data sets and TCGA-LIHC enrolled in the study.

Database	Source	Sample		Sample type	Sample backgrounds	Platform
		HCC	Non-tumor			
GSE14520	https://www.ncbi.nlm.nih.gov/geo/query/acc.cgi?acc=GSE14520	219	220	Liver	USA	Affymetrix U133A
GSE54236	https://www.ncbi.nlm.nih.gov/geo/query/acc.cgi?acc=GSE54236	81	80	Liver	Italy	Agilent G4112F
GSE87630	https://www.ncbi.nlm.nih.gov/geo/query/acc.cgi?acc=GSE87630	64	30	Liver	South Korea	Illumina HT-12
GSE109211	https://www.ncbi.nlm.nih.gov/geo/query/acc.cgi?acc=GSE109211	140	/	Liver	Spain	Illumina HT-12
TCGA-LIHC	https://portal.gdc.cancer.gov	352	/	Liver	USA	Illumina

To explore the potential biological roles of these 34 DEGs, Gene Ontology (GO) enrichment analysis were performed. As shown in Table S1, most genes were enriched in the biosynthetic process (such as carboxylic acid, organic acid, and monocarboxylic acid). These results suggested that the 34 genes were closely associated with HCC processing.

### Construction of an early relapse model from the GSE14520 cohort

Based on these 34 candidate genes and complete clinical prognostic information of GSE14520, the optimal cutoff and AUC values for predicting tumor early recurrence of each gene were determined by time-dependent ROC curve, and the *p-*values were determined by univariate Cox regression analysis, which could independently predict the tumor early recurrence (The optimal cutoff values and *p-*values were shown in Table S2). Finally, 15 DEGs with AUC > 0.55 and *p-*value < 0.25 were selected to construct the risk model using LASSO Cox Regression analysis.

**Figure 2:**
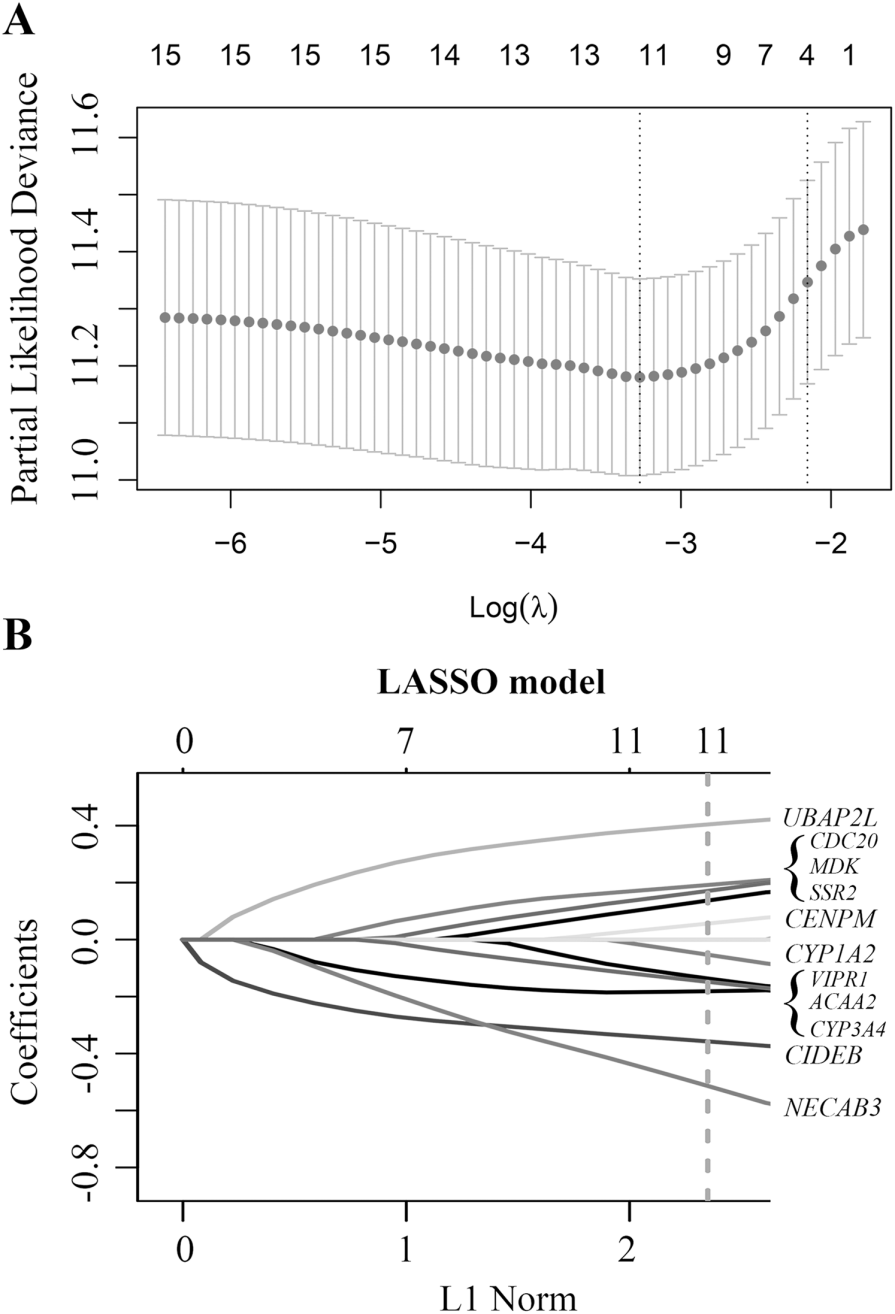
Construction of early relapse predictive model from GSE14520 cohort. (A) LASSO Cox analysis identified 11 DEGs most correlated with tumor recurrence in GSE14520 10-fold cross-validation for turning parameter selection in the LASSO model. (B) LASSO coefficient profiles of 11 DEGs, a vertical line at the value of 10-fold likelihood cross-validation was drawn.

Finally, a multi-mRNA model containing 11 genes with nonzero coefficients was generated at the lambda.min value (0.03786) chosen by 10-fold cross-validation and 100 times (Fig. 2A). These mRNAs included ubiquitin associated protein 2 like (*UBAP2L*), cell division cycle 20 (*CDC20*), midkine neurite growth-promoting factor 2 (*MDK*), signal sequence receptor subunit 2 (*SSR2*), centromere protein M (*CENPM*), cytochrome P450 family 1 subfamily A member 2 (*CYP1A2*), vasoactive intestinal peptide receptor 1 (*VIPR1*), acetyl-CoA acyltransferase 2 (*ACAA2*), cytochrome P450 family 3 subfamily A member 4 (*CYP3A4*), cell death-inducing DFFA-like effector b (*CIDEB*), and N-terminal EF-hand calcium-binding protein 3 (*NECAB3*) (Fig. 2B). The equation of 11-mRNA risk score model was: The model = (0.4044 * status of *UBAP2L*) + (0.1919 * status of *CDC20*) + (0.1714 * status of *MDK*) + (0.1375 * status of *SSR2*) + (0.0566 * status of *CENPM*) – (0.0538 * status of *CYP1A2*) – (0.1371 * status of *VIPR1*) – (0.1488 * status of *ACAA2*) – (0.1812 * status of *CYP3A4*) – (0.3578 * status of *CIDEB*) – (0.5150 * status of *NECAB3*).

### Evaluation and validation of the model in the discovery cohort

**Figure 3:**
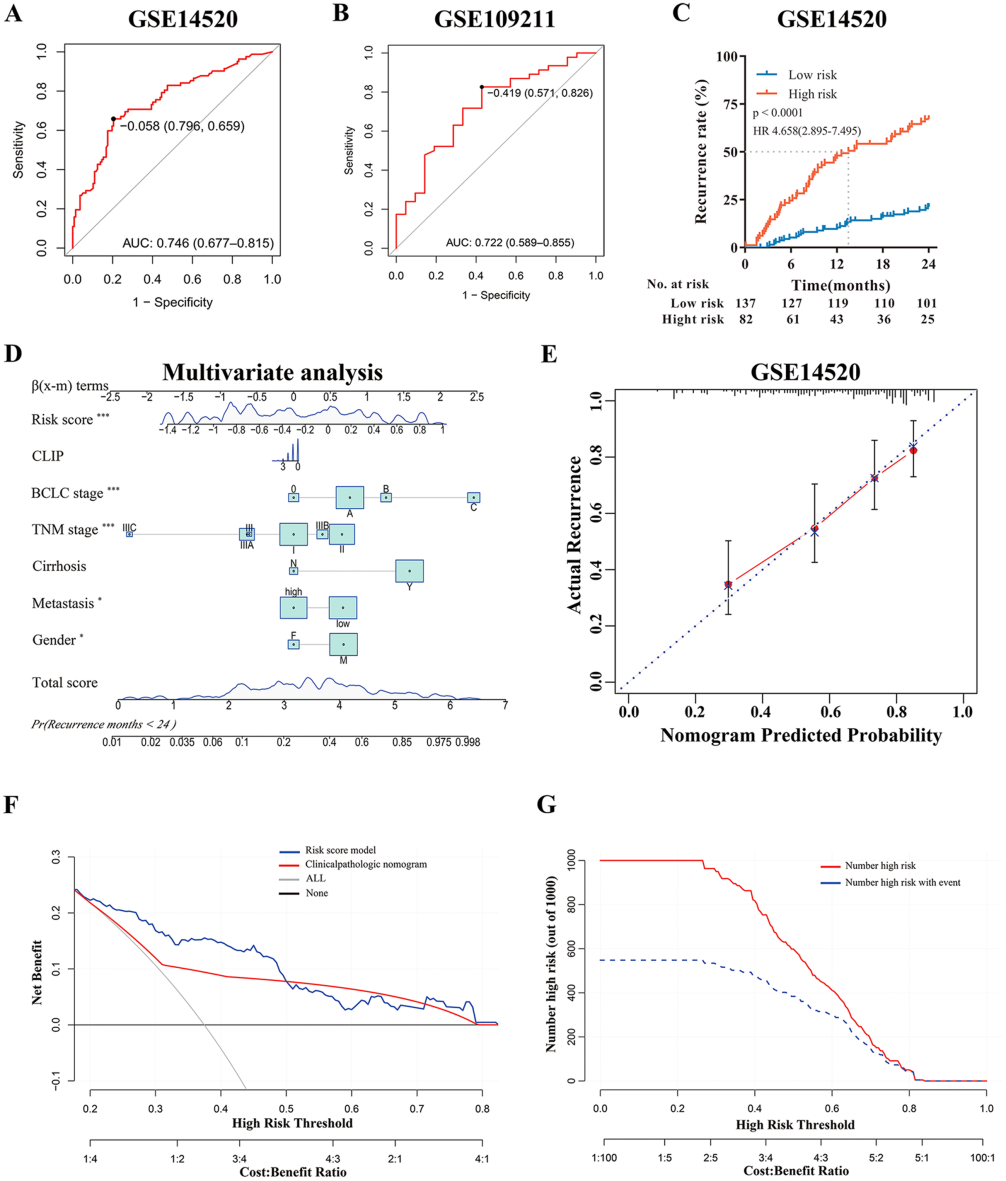
Evaluation and Validation of the risk model in discovery cohort. (A) Predictive value of time-dependent ROC curve for tumor recurrence at 24 months. (B) Predictive value of ROC curve for the response to adjuvant sorafenib. (C) Cumulative recurrence rates between high- or low- risk groups at the indicated time. (D) Nomogram for predicting cumulative risk of tumor recurrence at 24 months . Draw an upward vertical line from each variable to the ß(x-m) terms to calculate score. Based on cumulative sum, draw a downward vertical line from the total score line to calculate the probability of recurrence . The size of variable box represents the number of patients , and the height of the score curve represents the frequency. (E) Calibration curve of the risk score model in training cohorts. The bottom horizontal axis represents the predicted recurrence probability, and the left vertical axis represents the actual recurrence possibility; The dotted line of 45-degree represents the ideal prediction, and the red line represents the predictive performance of the risk score model. The closer the red line fit is to the 45-degree dotted line, the better prediction of the risk score model will be. The spikes along the top x-axis represents the distribution of recurrence probabilities predicted by model, and the height of the spikes represents the density of the predicted probabilities. (F) Decision curve analysis demonstrates the net benefit of the risk score model and clinicalpathologic nomogram in training cohorts. The horizontal axis was threshold probability, and the ordinate axis was the net benefit. The horizontal black line with a net benefit of zero indicates that no patients have recurrence, and the oblique gray line indicates that all patients have recurrence. (G) Clinical impact curve of the risk score model. Two horizontal axes show the correspondence between risk threshold and Cost:Benefit Ratio. Solid red line represents the total number of high-risk patients predicted by the risk model for each risk threshold, and dotted blue line represents the number of high-risk patients with recurrence under each threshold probability.

**Figure 4:**
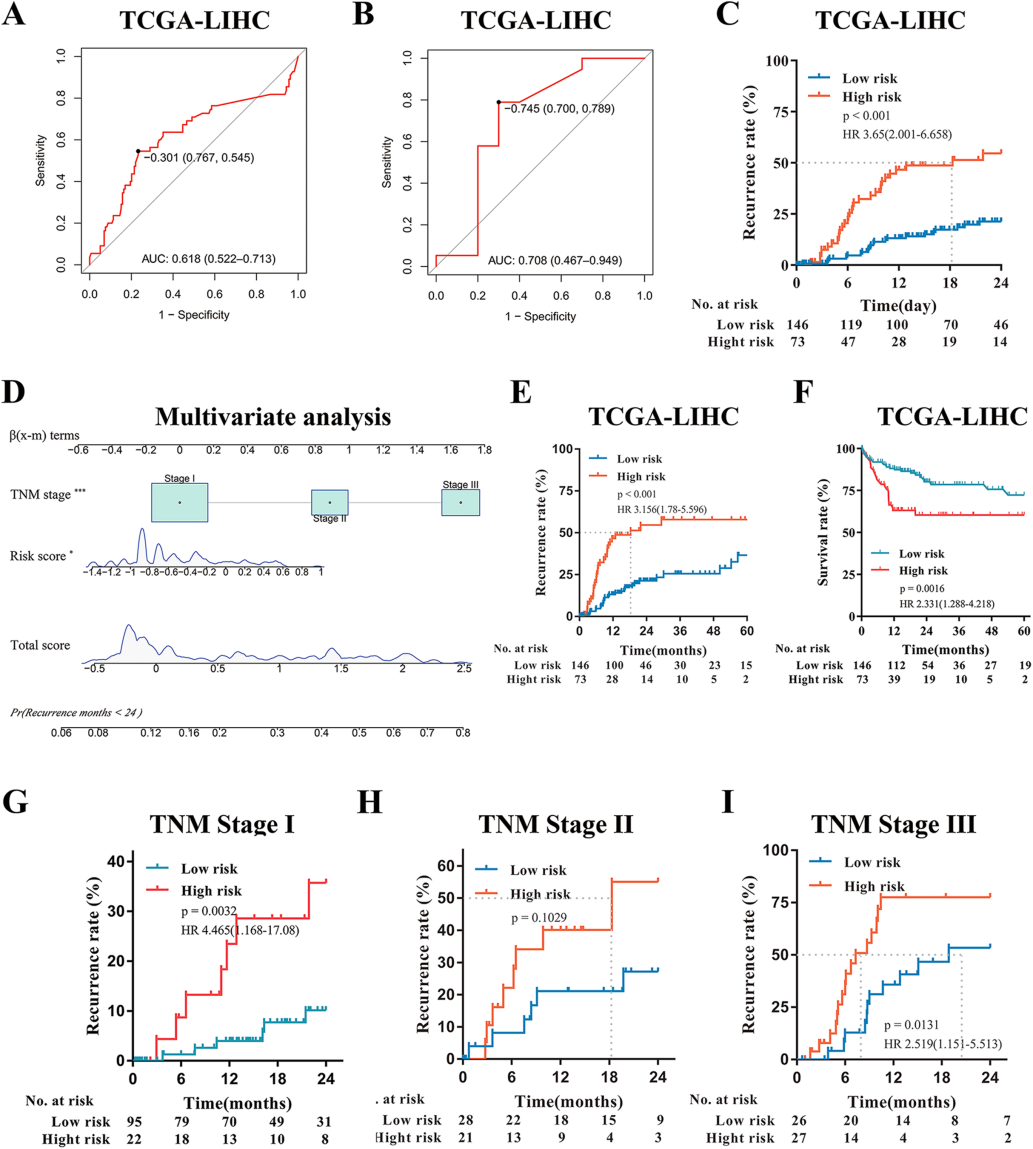
External Validation of the risk score model in TCGA-LIHC. (A) Predictive value of time-dependent ROC curve for recurrence at 24 month. (B) Predictive value of ROC curve for the response to adjuvant sorafenib. (C) Cumulative recurrence rates between high- or low- risk groups at the indicated time. (D) Nomogram for predicting cumulative risk of tumor recurrence at 24 months in TCGA cohorts. (E) Cumulative recurrence rate between high- or low- risk groups at the indicated time. (F) Kaplan–Meier survival analysis for overall survival at the indicated time. (G–I) Recurrence rate according to the risk score model classifier in subgroups of patients with TNM Stage I, II and III.

According to the multi-mRNA model, the score for each patient in GSE14520 and GSE109211 was calculated. The ROC curve showed that the AUC values of the score for predicting tumor early recurrence and response to sorafenib were 0.746 and 0.722, respectively (Figs. 3A and 3B), and the score of the sorafenib resistance was significantly higher than that of responders (*p* = 0.0024, Fig. 4B). The optimal cutoff value for relapse was –0.058, with a sensitivity of 0.659 and specificity of 0.796 (Fig. 3A). Based on this cutoff value (–0.058), we divided the patients in GSE14520 into two subgroups (high-risk group and low-risk group, Fig. S4A). Kaplan–Meier curves showed that patients with a high-risk score had worse recurrence rate (2-year: *p* < 0.0001, hazard ratio (HR): 4.658, confidence interval 95% CI [2.895–7.495]; 5-year: *p* < 0.0001, HR: 3.251, 95% CI [2.155–4.904]), and the median time to recurrence was 13.5 months (Fig. 3C and Fig. S2C). After adjusting by clinical factors (gender, metastasis, cirrhosis, TNM stage, BCLC stage, and CLIP stage) determined using univariate Cox regression analyses, the risk score model still had an excellent ability to independently predict the early recurrence of HCC, the risk model and other independent predictors were presented as a nomogram (Fig. 3D). Meanwhile, decision curve analysis (DCA) showed that if the threshold probability is higher than 0.2 and less than 0.5, the risk score model had a larger net benefit *versus* clinicopathologic nomogram (Fig. 3F). In addition, the DCA curve showed favorable agreement between risk model predicted recurrence probability and actual recurrence probability, and clinical impact curve also revealed that the patients could be significantly benefited from this model (Figs. 3E and 3G).

Furthermore, to explore the potential clinical value of the risk score model among patients with the same tumor stage category, Kaplan–Meier curves analysis was performed. For the GSE14520 cohort, when stratified by clinicopathologic features (BCLC staging and TNM staging), patients with a high-risk score displayed higher recurrence rates, especially those patients with tumors at the early stage (BCLC 0 and A, TNM I and II) (*p* < 0.001, Figs. S2A, S2B and S2D–S2F), and regardless the risk of tumor metastasis (all *p* < 0.0001, Figs. S2G and S2H). Even if the patients with advanced tumor stage (such as TNM Stage III) were excluded, the risk model still had high predictive value demonstrated by ROC (AUC value: 0.725, Fig. S2I). In addition, to verify the prognostic value of the risk model, we further conducted the time-dependent ROC curve for predicting survival and Kaplan–Meier curve analyses. It demonstrated that the AUC value was 0.715 (Fig. S3A) and patients with the high-risk score had remarkably worse survival rates (*p* < 0.0001, HR:3.811, 95% CI [2.365–6.142], Fig. S3B). Multivariate Cox regression analysis demonstrated that the risk model was still significantly related to survival (*p* < 0.001, Fig. S3C). Moreover, DCA revealed that the risk score model had a similar survival benefit to BCLC stage (Fig. S3D); and Calibration curve analysis showed the predicted probability survival value was consistent with actual survival (Fig. S3E). In conclusion, the risk score model was a reliable model with clinically and statistically significant for early-RFS patients and for predicting response to adjuvant sorafenib.

### External validation of the risk score model in TCGA-LIHC

Since the risk score model can be used to predict HCC recurrence and the response of patients to adjuvant sorafenib in the GSE14520 and GSE109211 cohort, then we further validated the predictive ability of the model in another HCC cohorts (TCGA-LIHC). According to the same formula, the risk score for each patient was calculated and the time-dependent ROC curve analysis was conducted. The AUC values of the model for predicting the tumor recurrence and response to adjuvant sorafenib were 0.618 (95% CI [0.522–0.713]) and 0.708 (95% CI [0.467–0.949]), respectively (Figs. 4A and 4B). Similar to the GSE14520 cohort, patients with high risk had a significantly higher recurrence rate and shorter median recurrence time (18.3 months) (all *p* < 0.001, Figs. 4C, 4E and Fig. S4C). And these sorafenib responders have a lower risk score (*p* = 0.0411, Fig. S4D). Moreover, patients with low risk had much better survival rate (*p* = 0.0016, HR: 2.331, 95% CI [1.288–4.218], Fig. 4F). Multivariate Cox regression analysis also demonstrated that the risk model was significantly related to the tumor recurrence (*p* < 0.05, Fig. 4D). In addition, Kaplan–Meier curves analysis was further performed in subsets of patients with different TNM stages. Interestingly, patients with a high-risk score also displayed higher recurrence rates in the validation cohorts with the different TNM stages (Figs. 4G–4I). In summary, the novel risk score model constructed using bioinformatic methods could effectively predict HCC early recurrence and the benefits from adjuvant sorafenib in patients with surgical resection.

## Discussion

Tumor recurrence is the major obstacle for long-term survival (Portolani et al., 2006; Han et al., 2019; Mahvi et al., 2018). For HCC, the recurrence rate after surgical resection is high (Llovet et al., 2016), and currently there is no universally accepted adjuvant therapy options or guidelines to prevent tumor recurrence (Kudo et al., 2015). Although some studies have demonstrated that the patients with hepatectomy would get clinical benefits from adjuvant therapy (Li et al., 2020a), it remains controversial (*e.g*. sorafenib *vs*. placebo, Bruix et al., 2015). Therefore, predicting tumor early recurrence and the favorable response to adjuvant therapy is an arduous and urgent task, which might help clinicians to evaluate the prognosis as early as possible and select patients who need adjuvant therapy.

In this study, we screened the commonly dysregulated genes related to tumor recurrence and response to adjuvant sorafenib in HCC (a total of 34 genes) and established a novel multi-mRNA predictive model for HCC recurrence and adjuvant sorafenib therapy by using univariate Cox regression analyses, time-dependent ROC curves analysis, and LASSO-COX regression model. This model could independently predict the early recurrence of HCC and the clinical benefits from adjuvant sorafenib, and its predictive ability was validated using another independent cohorts (TCGA-LIHC), showing a similar conclusion to that of the discovery cohorts in predicting HCC prognosis. The efficacy of this risk model was evaluated by the ROC curve, Kaplan–Meier curves, and DCA curves, showing a powerful predictive ability. Based on this risk score model, the prognosis and the response to adjuvant sorafenib of patients after liver resection could be easily predicted.

Notably, patients with poor clinical prognosis and resistance to adjuvant sorafenib have a higher risk score. Moreover, we found that the risk score, BCLC stage, TNM stage, metastasis, and gender could independently predict the early recurrence of HCC in discovery cohorts, while only risk score and TNM stage had this ability in validation cohorts. This indicates that our multi-mRNA model has universal applicability, and can provide a valuable reference for clinical decision-making. Besides, after stratification by clinicopathological features, our risk model was still a clinically applicable model in predicting early recurrence. These results again suggested that our model was superior to other clinical characters. In addition, multivariate analysis showed that patients with advanced tumor stage (TNM III stage) had a lower recurrence rate in the discovery cohorts, which might due to the high mortality of advanced patients. Lastly, the GO and KEGG analyses of these 34 genes indicated some acid biosynthetic processes and chemical carcinogenesis were significantly involved in HCC recurrence and response to sorafenib, but the potential mechanisms remain elusive and needed to be further studied.

Our risk model was composed of 11 genes, including five down-regulated genes (*CYP1A2*, *VIPR1*, *ACAA2*, *CYP3A4* and *CIDEB*) and six up-regulated genes (*UBAP2L*, *CDC20*, *MDK*, *SSR2, CENPM* and *NECAB3*). Most of these genes have been demonstrated to be linked with poor prognosis of tumor and the sensitivity of sorafenib. For example, as a tumor inhibitor, *CYP1A2* suppressed tumorigenicity by inhibiting HGF/MET and reduced the sensitivity of sorafenib *via* inhibiting the NF-kB signaling (Yu et al., 2021a, 2021b). In addition, *CYP3A4* is a main member of cytochrome P450 family of oxidizing enzymes, which is involved in the metabolism of drugs in liver (Karakurt, 2016). For example, CYP3A4 could mediate the metabolism of sorafenib in HCC cells (Ghassabian et al., 2012). Moreover, CYP3A4 metabolism results in formation of superoxide radicals, which may cause hepatotoxicity or carcinogenesis (Karakurt, 2016). *VIPR1* was closely related to the adverse prognosis of HCC (Lu et al., 2019). Kodama et al. revealed that *ACAA2* had particularly potent anti-proliferative activity, which is highly related to tumor growth (Kodama et al., 2016). Down-regulation of *NECAB3* in cancer cells was demonstrated to reduce tumorigenicity (Nakaoka et al., 2016), indicating the crucial role of NECAB3 in promoting cancer development. Up-regulation of *NECAB3* in cancer cells was demonstrated to enhance tumorigenesis (Nakaoka et al., 2016). *UBAP2L* promotes tumor growth and metastasis, and patients with high expression of *UBAP2L* have unfavorable prognoses (Li et al., 2018; Yoshida et al., 2020). *CDC20*, as a hub gene in HCC, plays a crucial role in tumor proliferation by governing *PHD3* protein (Shi et al., 2021). Overexpression of *MDK, CENPM*, and *SSR2* promotes tumor metastasis, angiogenesis, and tumorigenesis, leading to poor prognosis in patients with HCC (Gowhari Shabgah et al., 2021; Zheng et al., 2020; Xiao et al., 2019; Hong et al., 2020). Taken together, these genes, included in the risk model, are related to tumor recurrence and sorafenib resistance.

Although the risk model established in this study could provide basic data and potential use for clinicians to evaluate the risk of recurrence and the benefits from adjuvant sorafenib in patients, who received liver resection, it has some inevitable limitations. The transcriptional levels of gene abundance in our study are based on RNA-seq rather than qRT-PCR, but some studies have shown that the two methods are comparable (Zhou et al., 2019). Another important shortcoming is that our research data was downloaded from online databases and the model has not been validated with clinical patient specimens. Prospectively, clinical trials using conventional qRT-PCR methods and clinical patient specimens should be further performed to confirm the predictive value of the risk model.

## Conclusion

In conclusion, by using bioinformatic methods, we constructed a powerful risk model independent of clinicopathological variables for predicting tumor recurrence and response of patients to adjuvant sorafenib. Based on this model, clinicians could effectively evaluate the prognosis and the benefits of adjuvant sorafenib after liver resection, so could improve the strategies for personalized treatment accordingly. Furthermore, our study may provide a reference for further study to improve the prognosis of HCC patients with liver resection.

## Supplemental Information

10.7717/peerj.12554/supp-1 Supplemental Information 1 Significant gene annotations of GO enrichment analysis.

10.7717/peerj.12554/supp-2 Supplemental Information 2 ROC and Univariate Cox regression analysis in GSE14520 cohorts.

10.7717/peerj.12554/supp-3 Supplemental Information 3 Identification of genes associated with sorafenib. (A) Volcano plots of genes related to placebo in GSE109211 dataset. (B) Overlaping Analysis of sorafenib and placebo related genes in HCC.

10.7717/peerj.12554/supp-4 Supplemental Information 4 Stratification analyses of the risk score model based classifier. (A and B) Kaplan–Meier analyses of tumor recurrence rate in BCLC 0/ A and BCLC B/C subgroups. (C) Kaplan–Meier analysis of cumulative recurrence rate in patients with HCC. (D–F) Kaplan–Meier analysis of tumor recurrence rate according to the risk score model classifier in subgroups of patients with TNM Stage I, II and III. (G and H) Kaplan–Meier analyses of tumor recurrence rate in subgroups of patients with Low- or High- Metastasis. (I) Predictive value of ROC curve for recurrence at 24 months.

10.7717/peerj.12554/supp-5 Supplemental Information 5 Validation of risk score model for the prognostic value in GSE14520 cohorts. (A) Time-dependent ROC curves for overall survival. (B) Kaplan–Meier analysis of survival rate for patients with low- or high- risk. (C) Nomogram for predicting overall survival at 24 months in GSE14520 cohorts . (D) Decision curve analysis shows the net benefit of the risk score model and BCLC Staging for overall survival in training cohorts. (E) Calibration curve of the risk score model for predicting overall survival in GSE14520 cohorts .

10.7717/peerj.12554/supp-6 Supplemental Information 6 Distributions of risk score in different series. (A and C) Risk score analysis for patients in GSE14520 and TCGA-LIHC. (B and D) Distributions of risk score between response group and non-response group to adjuvant sorafenib.

## Additional Information and Declarations

### Data Availability

The following information was supplied regarding data availability: Data is available at NCBI GEO: GSE14520, GSE54236, GSE87630, and GSE109211. The TCGA-LIHC was the validation cohort downloaded from The Cancer Genome Atlas.
