# Supplementary material for: Prediction of early recurrence and response to adjuvant Sorafenib for hepatocellular carcinoma after resection
Source: PeerJ. 2021 Nov 26;9:e12554. doi: 10.7717/peerj.12554 (PMC8628622; doi:10.7717/peerj.12554)
Supplement: Supplemental Information 1 [file peerj-09-12554-s001.docx]

| Category | ID | Term | Count | p-Value |
| --- | --- | --- | --- | --- |
| GOTERM_BP_DIRECT | GO:0046394 | Carboxylic acid biosynthetic process | 6 | 0.000138 |
| GOTERM_BP_DIRECT | GO:0016053 | organic acid biosynthetic process | 6 | 0.00014 |
| GOTERM_BP_DIRECT | GO:0010821 | regulation of mitochondrion organization | 5 | 2.34E-05 |
| GOTERM_BP_DIRECT | GO:0072330 | monocarboxylic acid biosynthetic process | 5 | 0.000317 |
| GOTERM_BP_DIRECT | GO:0048285 | organelle fission | 5 | 0.001204 |
| GOTERM_CC_DIRECT | GO:0062023 | collagen-containing extracellular matrix | 5 | 0.000558 |
| GOTERM_CC_DIRECT | GO:0005811 | lipid droplet | 3 | 0.000316 |
| GOTERM_CC_DIRECT | GO:0005819 | spindle | 3 | 0.020386 |
| GOTERM_CC_DIRECT | GO:0005874 | microtubule | 3 | 0.033196 |
| GOTERM_MF_DIRECT | GO:0015631 | tubulin binding | 4 | 0.002709 |
| GOTERM_MF_DIRECT | GO:0008083 | growth factor activity | 3 | 0.002652 |
| GOTERM_MF_DIRECT | GO:0005201 | extracellular matrix structural constituent | 3 | 0.003765 |
| GOTERM_MF_DIRECT | GO:0050662 | coenzyme binding | 3 | 0.014801 |
| GOTERM_MF_DIRECT | GO:0005126 | cytokine receptor binding | 3 | 0.018604 |
