# Supplementary material for: Prediction of early recurrence and response to adjuvant Sorafenib for hepatocellular carcinoma after resection
Source: PeerJ. 2021 Nov 26;9:e12554. doi: 10.7717/peerj.12554 (PMC8628622; doi:10.7717/peerj.12554)
Supplement: Supplemental Information 2 [file peerj-09-12554-s002.docx]

| Candidate genes | ROC analysis | | | | | Univariate Cox |
| --- | --- | --- | --- | --- | --- | --- |
|  | AUC | 95%CI | Sensitivity | Specificity | Cutoff | p-value |
| CYP1A2 | 0.590 | 0.513-0.667 | 0.549 | 0.613 | 5.725 | 0.007 |
| CXCL12 | 0.532 | 0.453-0.610 | 0.817 | 0.270 | 7.161 | 0.138 |
| ABCA8 | 0.578 | 0.501-0.655 | 0.915 | 0.255 | 8.546 | 0.005 |
| VIPR1 | 0.611 | 0.533-0.688 | 0.256 | 0.912 | 3.774 | 0.007 |
| CYP3A4 | 0.574 | 0.495-0.652 | 0.756 | 0.416 | 8.548 | 0.007 |
| DCN | 0.510 | 0.432-0.587 | 0.854 | 0.255 | 6.682 | 0.227 |
| ECM1 | 0.504 | 0.426-0.582 | 0.780 | 0.292 | 5.354 | 0.326 |
| OGDHL | 0.580 | 0.502-0.659 | 0.561 | 0.577 | 6.251 | 0.314 |
| CYP3A43 | 0.624 | 0.548-0.701 | 0.671 | 0.577 | 5.482 | 0.001 |
| IGFBP3 | 0.555 | 0.478-0.632 | 0.890 | 0.241 | 7.931 | 0.308 |
| CIDEB | 0.612 | 0.537-0.687 | 0.707 | 0.562 | 8.804 | 0.000 |
| LUM | 0.480 | 0.402-0.558 | 0.927 | 0.139 | 9.303 | 0.172 |
| RBP1 | 0.467 | 0.388-0.546 | 0.598 | 0.416 | 6.619 | 0.749 |
| ACAA2 | 0.607 | 0.529-0.684 | 0.463 | 0.730 | 8.808 | 0.001 |
| GABARAPL1 | 0.498 | 0.419-0.577 | 0.561 | 0.518 | 6.254 | 0.137 |
| PHGDH | 0.548 | 0.470-0.627 | 0.780 | 0.350 | 5.643 | 0.060 |
| STARD5 | 0.566 | 0.489-0.644 | 0.841 | 0.321 | 5.007 | 0.026 |
| PPARGC1A | 0.564 | 0.482-0.646 | 0.451 | 0.730 | 4.905 | 0.003 |
| ADAMTSL2 | 0.503 | 0.425-0.582 | 0.829 | 0.248 | 4.247 | 0.158 |
| NTF3 | 0.513 | 0.432-0.593 | 0.573 | 0.511 | 4.127 | 0.231 |
| CENPE | 0.552 | 0.473-0.631 | 0.622 | 0.504 | 4.326 | 0.034 |
| STXBP6 | 0.480 | 0.400-0.560 | 0.024 | 1.000 | 6.765 | 0.009 |
| GPAA1 | 0.588 | 0.509-0.668 | 0.549 | 0.672 | 7.474 | 0.402 |
| CENPM | 0.605 | 0.526-0.683 | 0.598 | 0.577 | 4.585 | 0.004 |
| LAGE3 | 0.531 | 0.453-0.610 | 0.476 | 0.613 | 6.723 | 0.245 |
| NECAB3 | 0.581 | 0.504-0.658 | 0.829 | 0.416 | 6.047 | 0.001 |
| CDKN2A | 0.534 | 0.454-0.614 | 0.378 | 0.745 | 5.670 | 0.036 |
| TARBP1 | 0.562 | 0.481-0.642 | 0.256 | 0.883 | 6.200 | 0.539 |
| SSR2 | 0.588 | 0.510-0.666 | 0.683 | 0.518 | 9.082 | 0.003 |
| UBAP2L | 0.631 | 0.554-0.707 | 0.476 | 0.745 | 7.809 | 0.001 |
| TPX2 | 0.543 | 0.464-0.621 | 0.476 | 0.657 | 6.513 | 0.013 |
| TNRD1 | 0.546 | 0.467-0.624 | 0.707 | 0.423 | 8.701 | 0.090 |
| CDC20 | 0.582 | 0.505-0.660 | 0.549 | 0.642 | 5.729 | 0.001 |
| MDK | 0.589 | 0.512-0.665 | 0.768 | 0.431 | 6.109 | 0.004 |
