# Supplementary figures and images for: Prediction of early recurrence and response to adjuvant Sorafenib for hepatocellular carcinoma after resection
Source: PeerJ. 2021 Nov 26;9:e12554. doi: 10.7717/peerj.12554 (PMC8628622; doi:10.7717/peerj.12554)

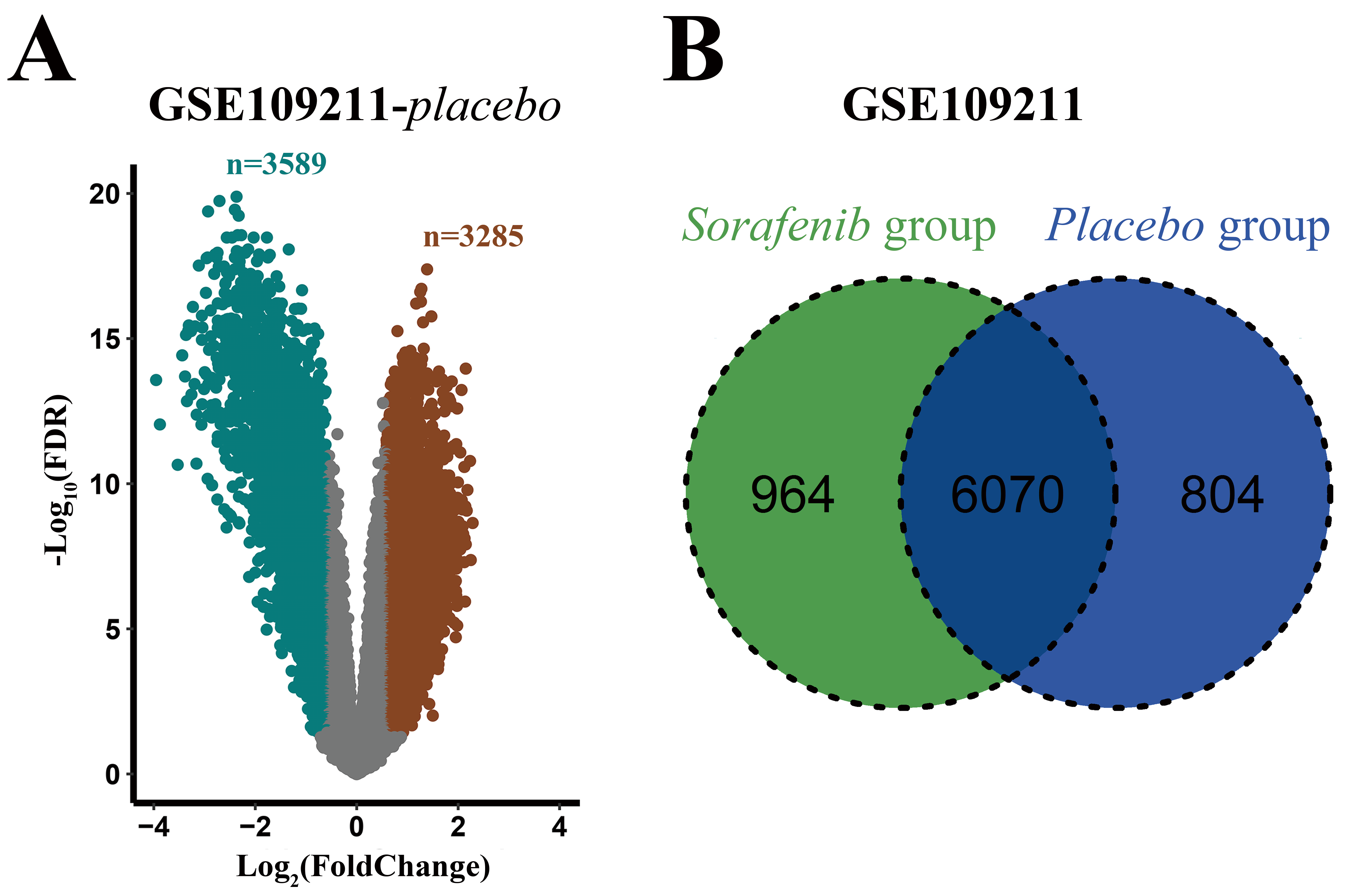

Supplement: Supplemental Information 3 — (A) Volcano plots of genes related to placebo in GSE109211 dataset. (B) Overlaping Analysis of sorafenib and placebo related genes in HCC. [file peerj-09-12554-s003.png]

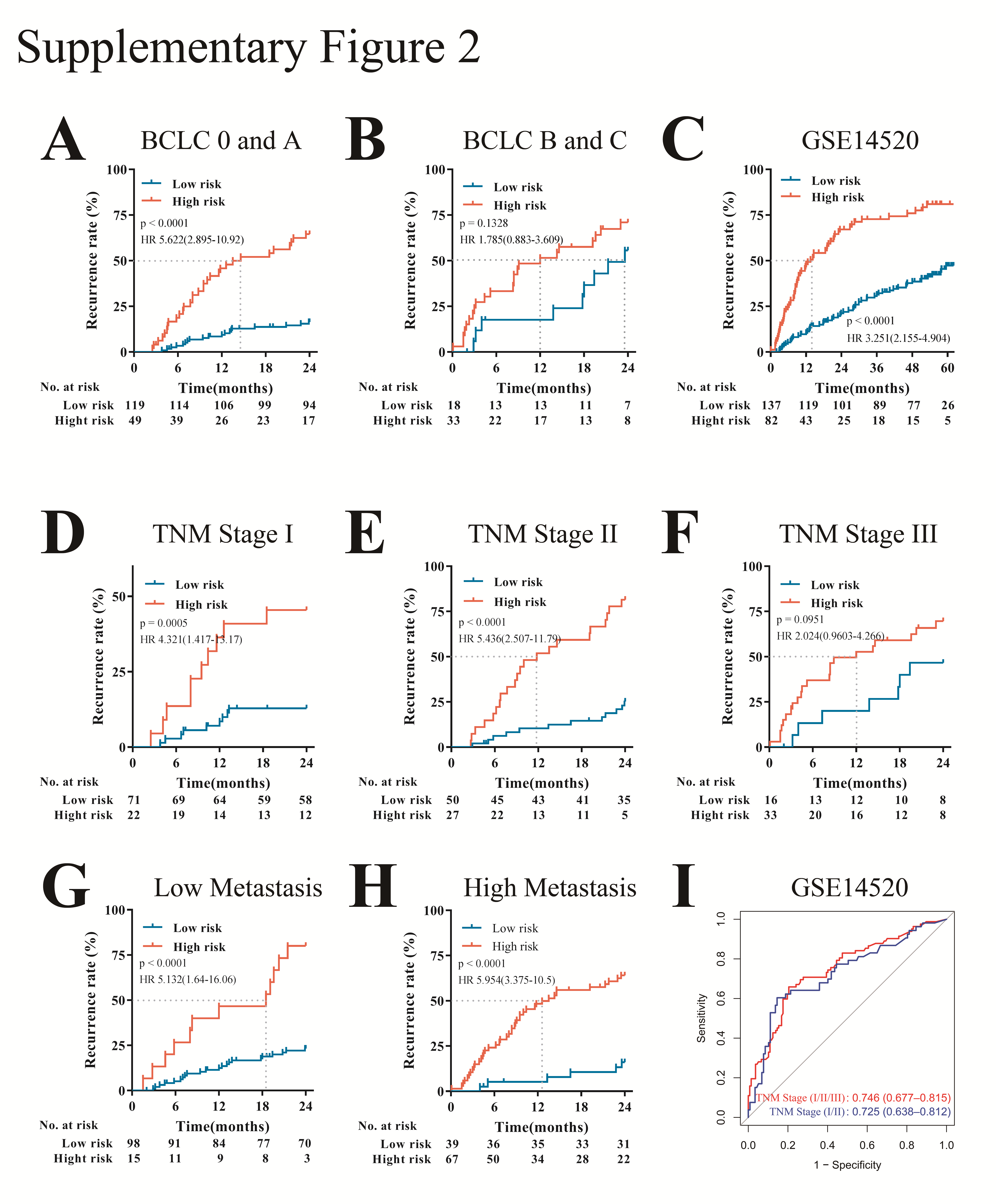

Supplement: Supplemental Information 4 — (A and B) Kaplan–Meier analyses of tumor recurrence rate in BCLC 0/ A and BCLC B/C subgroups. (C) Kaplan–Meier analysis of cumulative recurrence rate in patients with HCC. (D–F) Kaplan–Meier analysis of tumor recurrence rate according to the risk score model classifier in subgroups of patients with TNM Stage I, II and III. (G and H) Kaplan–Meier analyses of tumor recurrence rate in subgroups of patients with Low- or High- Metastasis. (I) Predictive value of ROC curve for recurrence at 24 months. [file peerj-09-12554-s004.png]

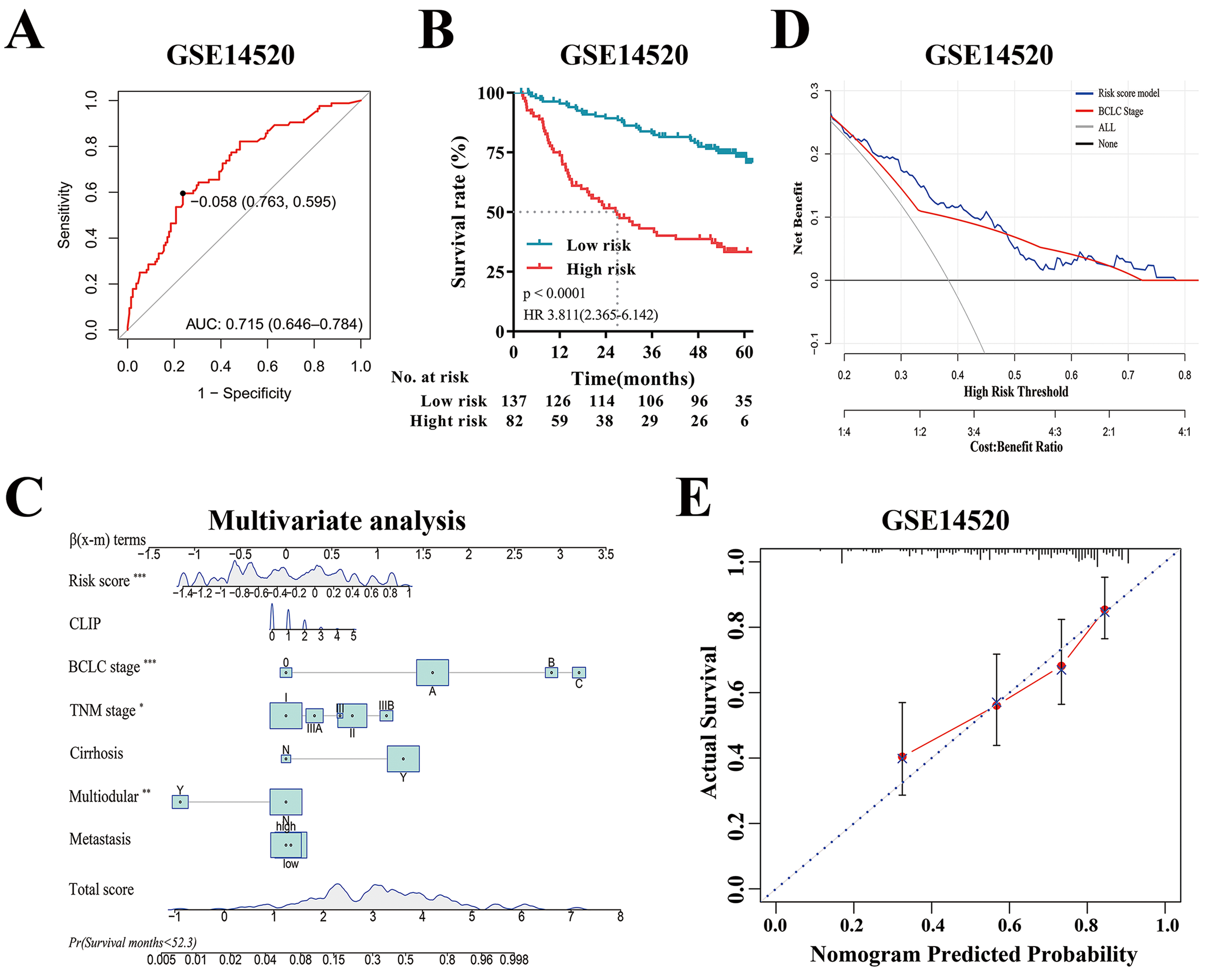

Supplement: Supplemental Information 5 — (A) Time-dependent ROC curves for overall survival. (B) Kaplan–Meier analysis of survival rate for patients with low- or high- risk. (C) Nomogram for predicting overall survival at 24 months in GSE14520 cohorts . (D) Decision curve analysis shows the net benefit of the risk score model and BCLC Staging for overall survival in training cohorts. (E) Calibration curve of the risk score model for predicting overall survival in GSE14520 cohorts . [file peerj-09-12554-s005.png]

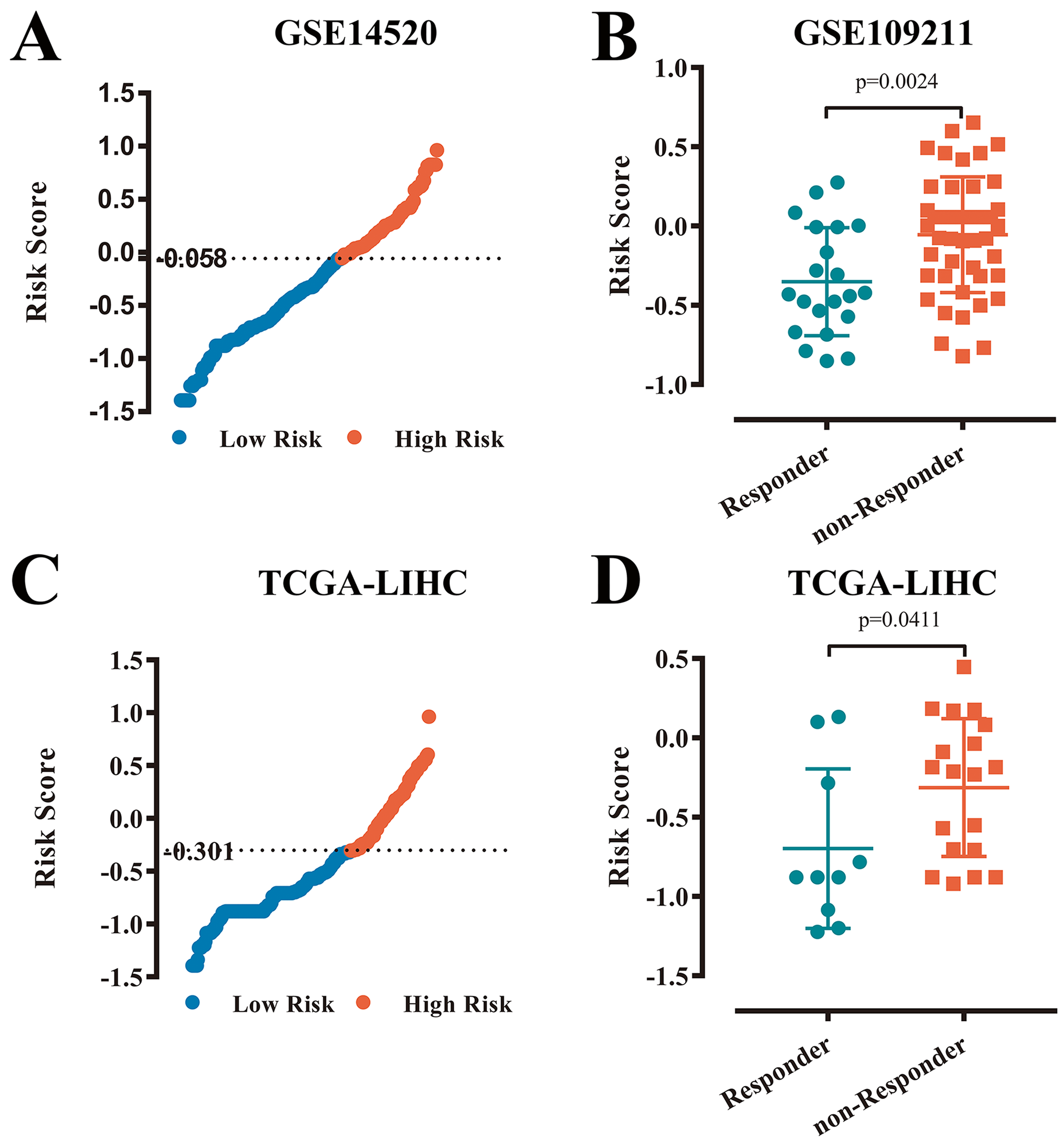

Supplement: Supplemental Information 6 — (A and C) Risk score analysis for patients in GSE14520 and TCGA-LIHC. (B and D) Distributions of risk score between response group and non-response group to adjuvant sorafenib. [file peerj-09-12554-s006.png]
